# Renal Injury during Long-Term Crizotinib Therapy

**DOI:** 10.3390/ijms19102902

**Published:** 2018-09-25

**Authors:** Taro Yasuma, Tetsu Kobayashi, Corina N. D’Alessandro-Gabazza, Hajime Fujimoto, Kentaro Ito, Yoichi Nishii, Kota Nishihama, Prince Baffour Tonto, Atsuro Takeshita, Masaaki Toda, Esteban C. Gabazza, Osamu Taguchi, Shigenori Yonemura, Osamu Hataji

**Affiliations:** 1 Department of Immunology, Faculty and Graduate School of Medicine, Mie University, Edobashi 2-174, Tsu, Mie 514-8507, Japan; t-yasuma0630@clin.medic.mie-u.ac.jp (T.Y.); 316MS02@m.mie-u.ac.jp (P.B.T.); johnpaul0114@yahoo.co.jp (A.T.); t-masa@doc.medic.mie-u.ac.jp (M.T.); gabazza@doc.medic.mie-u.ac.jp (E.C.G.); 2 Department of Diabetes and Endocrinology, Faculty and Graduate School of Medicine, Mie University, Edobashi 2-174, Tsu, Mie 514-8507, Japan; kn2480@gmail.com; 3 Department of Pulmonary and Critical Care Medicine, Faculty and Graduate School of Medicine, Mie University, Edobashi 2-174, Tsu, Mie 514-8507, Japan; kobayashitetsu@hotmail.com (T.K.); genfujimoto1974@yahoo.co.jp (H.F.); 4 Respiratory Center, Matsusaka Municipal Hospital, Tonomachi 1550, Matsusaka, Mie 515-8544, Japan; kentarou_i_0214@yahoo.co.jp (K.I.); mchnishii@city-hosp.matsusaka.mie.jp (Y.N.); taguchio@clin.medic.mie-u.ac.jp (O.T.); mch1031@city-hosp.matsusaka.mie.jp (O.H.); 5 Department of Nephrology, Matsusaka Municipal Hospital, Tonomachi 1550, Matsusaka, Mie 515-8544, Japan; amikurumika@yahoo.co.jp

**Keywords:** lung cancer, renal injury, fibrosis, crizotinib, anaplastic lymphoma kinase, cystic formation

## Abstract

Crizotinib is highly effective against anaplastic lymphoma kinase-positive and c-ros oncogen1-positive non-small cell lung cancer. Renal dysfunction is associated with crizotinib therapy but the mechanism is unknown. Here, we report a case of anaplastic lymphoma kinase positive non-small cell lung cancer showing multiple cysts and dysfunction of the kidneys during crizotinib administration. We also present results demonstrating that long-term crizotinib treatment induces fibrosis and dysfunction of the kidneys by activating the tumor necrosis factor-α/nuclear factor-κB signaling pathway. In conclusion, this study shows the renal detrimental effects of crizotinib, suggesting the need of careful monitoring of renal function during crizotinib therapy.

## 1. Introduction

Crizotinib is a selective inhibitor of several receptor tyrosine kinases including anaplastic lymphoma kinase (ALK), hepatocyte growth factor receptor (HGF receptor, proto-oncogene c-Met) and c-ros oncogene 1 (ROS1) [1,2]. *ALK* rearrangements are found in approximately 5% of patients with non–small cell lung cancer (NSCLC) [3,4,5]. Crizotinib is highly effective against *ALK*-positive and *ROS1*-positive NSCLC and its clinical use has been approved in many countries [6,7,8]. The Food and Drug Administration of United States approved the clinical use of crizotinib in 2011 for *ALK*-positive NSCLC and in 2016 for *ROS1*-positive NSCLC. However, several adverse effects such as gastrointestinal complaints, visual disturbances and interstitial lung disease have been reported during clinical trials of crizotinib [8,9]. Renal cysts and functional impairment of the kidneys have also been reported in patients treated with crizotinib [10,11,12,13,14]. However, the underlying mechanism of renal complication associated with crizotinib therapy is unknown.

In this study, we reported a case of *ALK*-positive NSCLC with multiple renal cysts and renal dysfunction during crizotinib therapy and described the functional and pathological changes observed after long-term administration of crizotinib in an experimental mouse model.

## 2. Results

### 2.1. Case Report

**Figure 1:**
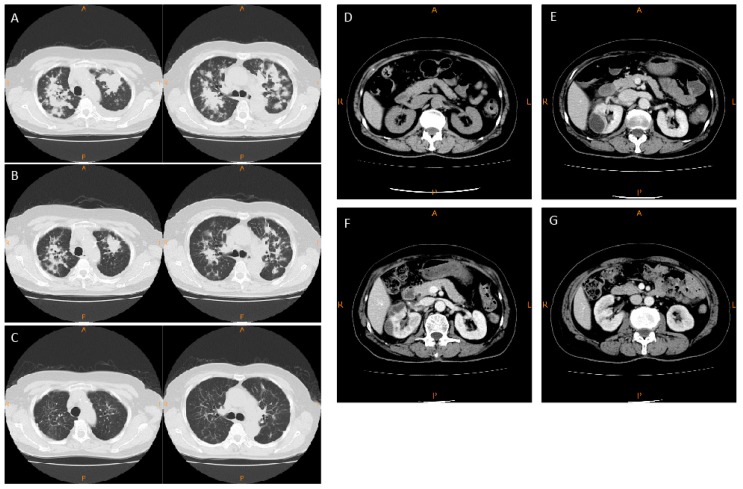
Chest and abdominal computed tomography (CT) in the present case. Chest CT of the patient with *ALK*-positive NSCLC at diagnosis (**A**), after 1 month (**B**), and after 11 months (**C**) of crizotinib therapy. Abdominal CT of the patient before therapy with crizotinib (**D**), 11 months after crizotinib therapy (**E**), 13 months (**F**) after crizotinib therapy, and after stopping the drug (**G**). NSCLC: non–small cell lung cancer; *ALK*: anaplastic lymphoma kinase.

A 71-year-old woman consulted the Respiratory Center of Matsusaka Municipal Hospital. The patient was being treated with amlodipine because of arterial hypertension. Lung adenocarcinoma with *ALK* arrangement was diagnosed based on clinical and pathological findings. Therapy with crizotinib (500 mg/day) was associated with marked tumor shrinkage and clinical improvement (Figure 1A–C). Parameters of kidney function were normal before the initiation of crizotinib. Three weeks following crizotinib administration, the blood level of creatinine increased from 0.73 mg/dL (pre-treatment value) to 1.21 mg/dL and remained at similar levels thereafter, but there were no abnormal findings in the kidneys upon computed tomography CT (Figure 1D). Eleven months after starting crizotinib treatment, the blood level of creatinine increased further (1.68 mg/dL) and multiple (>3) renal cysts were detected by CT examination (Figure 1E). Multiseptated renal cysts were detected by CT thirteen months after initiation of crizotinib (Figure 1F). Ultrasound study showed cystic formations, normal renal size and normal blood flow in the kidneys. Laboratory analysis of the cream-colored liquid obtained by ultrasound-guided cyst aspiration showed no cancer cells and microbial culture was negative. Urine analysis showed a mild proteinuria. Crizotinib was stopped and alectinib was started instead for the control of lung tumor. The blood level of creatinine decreased to 0.86 mg/dL after three weeks and the renal cysts regressed after three months of crizotinib withdrawal (Figure 1G). 

### 2.2. Experimental Animal Model

Pre-existing renal cysts enlarged during crizotinib administration in mice.

**Figure 2:**
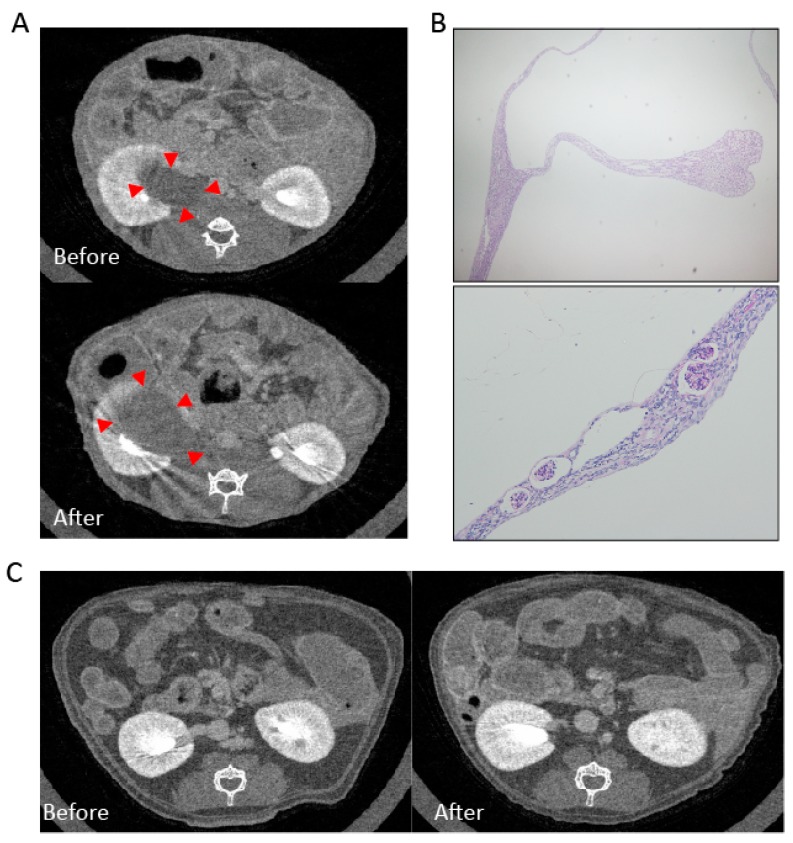
Enlargement of pre-existing cyst after crizotinib administration. Micro-CT images of a mouse show enlargement of kidney cyst after crizotinib treatment (**A**) red arrowheads. Periodic acid–Schiff staining showed compressed glomeruli and tubules (**B**) upper panel at ×40 and lower panel at ×100 magnification. The micro-CT of other mice with no pre-existing cyst show no cystic formation after crizotinib (**C**).

We performed an in vivo experiment to evaluate the long-term effect of crizotinib on renal function and pathology. Mice were allocated to a control group and a crizotinib-treated group. Mice of the crizotinib-treated group received long-term crizotinib administration. To assess the renal morphological changes, we performed micro-CT of kidneys before and after crizotinib treatment. Upon micro-CT scanning, one mouse showed a preexisting cystic lesion that enlarged during crizotinib administration (Figure 2A). The volume of the cyst as evaluated by contrast computed tomography increased from 0.51 cm^3^ before treatment to 0.72 cm^3^ after treatment. Periodic acid–Schiff staining of the cyst showed empty cysts with compressed renal parenchymal structures (Figure 2B). Apart from this mouse, no other mouse showed cystic formation in the kidneys before or after crizotinib treatment (Figure 2C).

#### 2.2.1. Mesangial Expansion in Crizotinib-Treated Mouse

**Figure 3:**
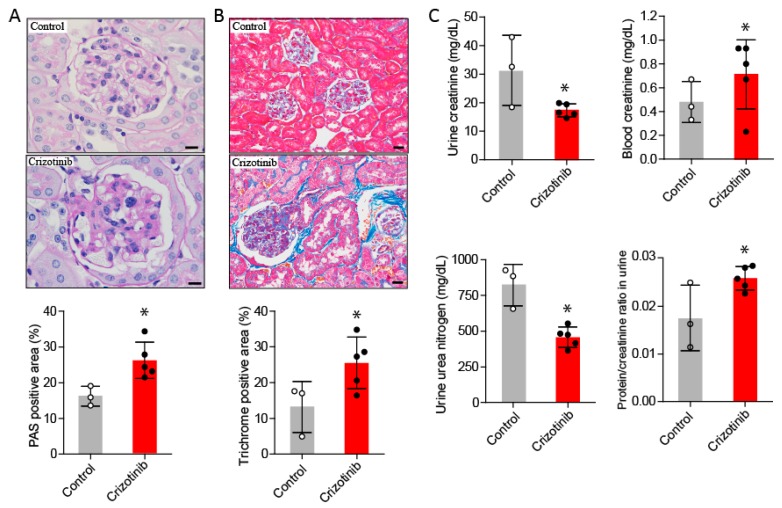
Renal fibrosis and increased markers of renal failure after crizotinib administration. Periodic acid-Schiff staining shows glomerular mesangial expansion in mice treated with crizotinib compared to untreated mice (**A**), scale bar indicates 10 µm. Masson’s trichrome staining shows increased glomerular and interstitial fibrosis in the kidneys from mice treated with crizotinib compared to those from untreated mice (**B**), scale bar indicates 20 µm. The blood level of creatinine, urine levels of urea nitrogen and creatinine and the ratio of total protein to creatinine in urine were significantly different between control and crizotinib groups (**C**). Data are mean ± SD. Control group *n* = 3; crizotinib group *n* = 5. * *p* < 0.05 versus control group.

Enhanced deposition of periodic acid-Schiff (PAS) positive substances was observed in glomeruli from the crizotinib-treated mice compared to untreated control mice (Figure 3A).

#### 2.2.2. Crizotinib Caused Renal Histopathological Changes

Increased staining for collagen in glomerular and renal interstitial areas was observed in mice treated with crizotinib compared to untreated mice (Figure 3B).

#### 2.2.3. Crizotinib Impaired Renal Function

Compared to the control mice, the plasma concentration of creatinine and the ratio of urine total protein to creatinine were significantly increased in the crizotinib-treated mice. Furthermore, urine concentration of creatinine and urea nitrogen were significantly decreased in the crizotinib-treated mice compared to control mice (Figure 3C).

### 2.3. Crizotinib Associated with Enhanced Inflammatory Markers in the Kidneys

**Figure 4:**
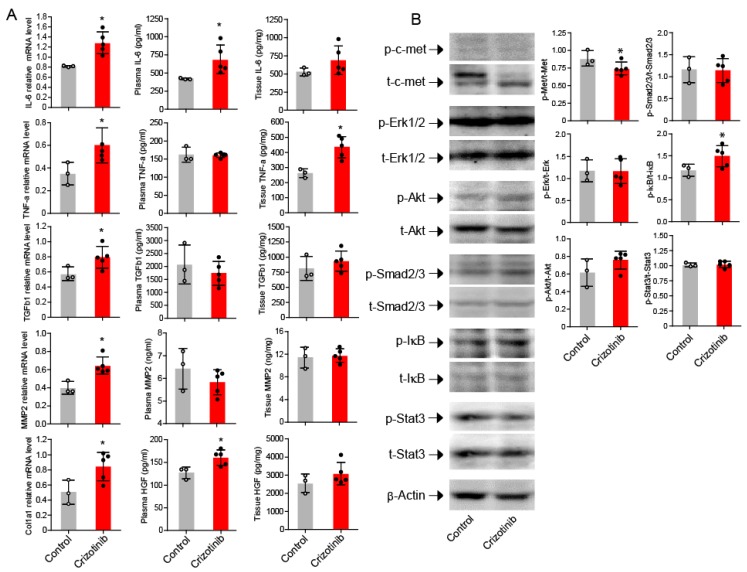
Cytokines, proteases and signal pathways after crizotinib administration. Increased mRNA expression of Col1a1, TGFβ1, IL-6, TNFα, and MMP2 in mice treated with crizotinib compared to untreated mice (**A**). Significant difference in phosphorylation level of c-Met and IκB between mice treated with and without crizotinib (**B**). Data are mean ± SD. Control group *n* = 3; crizotinib group *n* = 5. * *p* < 0.05 versus control group.

The relative mRNA expressions of IL-6, TNFα, TGFβ1, MMP2, and collagen I were increased in mice treated with crizotinib compared to untreated mice (Figure 4A). The plasma concentrations of IL-6 and HGF, and the kidney tissue levels of TNFα were also significantly increased in mice treated with crizotinib compared to control mice (Figure 4A).

### 2.4. Activation of NF-κB in the Kidneys after Crizotinib Therapy

As expected, c-Met activation was significantly decreased in the kidneys from mice treated with crizotinib compared to untreated mice (Figure 4B). Phosphorylated IκB was significantly increased in mice treated with crizotinib compared to untreated counterparts, but there was no significant difference in phosphorylation of Erk, Akt, Smad2/3, or Stat3 between treated and untreated mice (Figure 4B).

## 3. Discussion

The development of complex renal cysts associated with crizotinib treatment has been previously documented [10,11,12,13,14]. In a retrospective study among thirty-two Taiwanese patients with ALK-positive NSCLC treated with crizotinib, seven patients presented renal cysts that regressed after drug withdrawal [12]. In another retrospective analysis among seventeen patients with renal cysts associated with crizotinib treatment, seven patients showed compression of adjacent structures by cystic growth although the majority of patients were asymptomatic [13]. The evolution pattern of renal cysts during crizotinib treatment is variable but most renal cysts are asymptomatic, enlarge or spontaneously regress without crizotinib withdrawal [12,15,16,17]. In some instances the cysts regress after drug discontinuation [18]. Here, we also showed a case of ALK-positive non-small cell lung cancer with multiple renal cysts that developed during crizotinib administration. Although this case report is not the first, it is presented here to further illustrate the relevance of this treatment-related adverse effect in clinical practice and to emphasize the urgent need to clarify the mechanistic pathway.

The mechanistic pathways leading to cystic formation and renal dysfunction during crizotinib therapy remain unknown. A previous study showed that hepatocyte growth factor (HGF) and its receptor c-Met promote cystogenesis [19]. HGF-mediated activation of Mapk/Erk and/or Stat3 appears to be an important mediator of cystic formation [20,21,22]. Crizotinib inhibits c-Met and thus the involvement of c-Met in drug action would be paradoxical. Here, we confirmed inhibition of c-Met by crizotinib, but found no significant activation of Mapk/Erk or Stat3 signal pathway in mice treated with crizotinib. This suggests that alternative mechanisms may be involved in crizotinib-mediated cystogenesis in the kidneys. It is worth noting that, in the current report, the renal cyst of the patient worsened during crizotinib therapy and that the pre-existing renal cyst of a mouse enlarged after long-term administration of the drug. Based on these findings, it is reasonable to speculate that renal cysts develop only in subjects with pre-existing renal cysts that subsequently enlarge and become radiologically detectable during crizotinib administration. In our study, mice receiving crizotinib therapy showed no new development of cysts. The lack of cystic formation in our present experimental mouse model may be explained by too little exposure to the drug or the absence of a species-dependent propensity for developing the disease.

Patients with NSCLC may also have lower estimated glomerular filtration rates or dysfunction of the kidneys during crizotinib therapy that improves after drug withdrawal [23,24,25]. Patients with NSCLC and renal dysfunction before crizotinib administration have impaired renal function if they are treated with crizotinib [26]. The cause of the renal dysfunction is unknown. Renal biopsy performed in one case during the acute phase of the kidney dysfunction disclosed histopathological findings of mesangiolysis and acute tubular necrosis, but there is no report of biopsy findings in the chronic phase of the disease [25]. Our results are consistent with these observations. Here, we showed that mice receiving crizotinib over the long-term have renal dysfunction as demonstrated by high blood levels of creatinine, elevated urine total protein to creatinine ratio, as well as low levels of urine creatinine and urine urea nitrogen. Interestingly, PCR analysis showed high mRNA expression of collagen I and the histopathological study disclosed abnormal glomerular mesangial expansion and increased interstitial collagen deposition in the kidneys of mice treated with crizotinib compared to untreated counterparts, suggesting a pro-fibrotic activity of crizotinib in the kidneys.

Fibrosis and impaired dysfunction of the kidneys during crizotinib therapy may be explained by blockade of the protective and anti-fibrotic activity of the HGF/c-Met pathway in the kidneys. The HGF/c-Met signaling pathway is known to promote: (1) inhibition of renal interstitial myofibroblasts by intercepting Smad2/3 signal transduction; (2) reduction of TGFβ1-mediated proliferation; (3) differentiation and secretory activity of fibroblasts; and (4) amelioration of podocyte injury and proteinuria [27,28,29,30,31,32,33]. Here we found no changes in Smad2/3 phosphorylation in mice treated with crizotinib compared to untreated mice. In addition, Akt phosphorylation, which may promote fibrosis by inhibiting apoptosis of myofibroblasts, remained unaffected in the kidneys after crizotinib administration [34]. However, we found increased activation of the NF-κB pathway as demonstrated by the increased p-IκB/t-IκB ratio in association with decreased c-Met phosphorylation, as well as increased levels of TNFα and IL-6 in crizotinib-treated mice compared to control counterparts. TNF family cytokines can activate the NF-κB signaling pathway and NF-κB activation can induce collagen expression and cause tissue fibrosis in a TGFβ-independent fashion [35,36,37]. The NF-κB pathway may also cause tissue fibrosis by promoting differentiation of epithelial cells to fibroblasts [38,39]. The HGF/c-Met axis has been reported to decrease activation of NF-κB [40,41,42]. Therefore, it is conceivable that inhibition of c-Met by crizotinib causes renal injury and subsequent fibrosis by triggering activation of the NF-κB pathway [43]. However, the potential role of other TGFβ/Smad-independent pathways in crizotinib-associated renal fibrosis should also be evaluated in future studies [44].

The report of only one case, the small number of mice used in the experimental study and the fact that de novo cystic formation was not observed after crizotinib administration are limitations of the present study.

In brief, this study provides new evidence on possible mechanistic pathways causing morphological abnormalities and dysfunction of the kidneys in patients with lung cancer treated with crizotinib.

## 4. Materials and Methods

### 4.1. Experimental Animal Model

Wild-type C57BL/6 male mice (8 to 10 weeks old) weighing 19 to 22 g were used in the experiment. Mice were maintained in a specific pathogen-free environment under a 12 h light/dark cycle in the animal house of Mie University. Mice were allocated to a control group (*n* = 3) and a crizotinib-treated group *(n* = 5). The dose of crizotinib prescribed to patients with cancer is usually 400 to 500 mg (6–7 mg/kg) per day and therapy is generally continued for several months or years as long as the drug is beneficial to the patient [7]. In experimental animals, crizotinib has shown effective anti-tumor activity at doses of 10, 25 or 100 mg/kg/day after 4 or more weeks of treatment [2,45,46]. In the present study, to ensure chronic exposure to the drug, we treated mice with crizotinib by oral administration at a dose of 25 mg/kg/day for a period of 50 days. Mice were sacrificed on day 51 after the initial treatment.

### 4.2. Micro CT of Kidneys

Contrast-enhanced micro-CT of kidneys was performed with an X-ray CT system (Latheta LCT-200, Hitachi Aloka Medical Ltd., Tokyo, Japan) before crizotinib treatment started and 47 days after crizotinib treatment began. Under anesthesia with isoflurane, mice received an intravenous infusion of Iohexol (Daiichi-Sankyou, Tokyo, Japan), an iodine contrast medium, at a dose of 10 mL/kg before CT scanning. CT scanning was performed under conditions previously described [47]. Quantitative assessment of the lesion area was performed using the La Theta software version 3.30 (Hitachi-Aloka Medical Ltd., Tokyo, Japan).

### 4.3. Mouse Sacrifice and Sampling

Euthanasia of the experimental animals was performed using an overdose of intraperitoneal pentobarbital. Samples for biochemical and histological examinations were subsequently taken. Blood sampling was carried out by closed-chest heart puncture and samples were collected in tubes containing 10 U/mL heparin. Urine spot collection was also done for biochemical analysis.

### 4.4. Biochemical Analysis

Plasma and urine creatinine levels were measured by Jaffe’s reaction (Creatinine Companion Kit; Exocell, Philadelphia, PA, USA) and the concentration of total protein was measured using a dye-binding assay (BCA^TM^ protein assay kit; Pierce, Rockford, IL, USA). Urea nitrogen was measured by colorimetric method (NCal^TM^ NIST-Calibrated Kit; Arbor Assays, Ann Arbor, MI, USA) according to the manufacturer’s instructions. The concentrations of interleukin (IL)-6 and tumor necrosis factor (TNF)-α were measured using enzyme immunoassay kits from BD Biosciences (Tokyo, Japan). The concentrations of transforming growth factor (TGF)-β1, metalloproteinase (MMP)-2 and hepatocyte growth factor (HGF) were measured using a commercial enzyme immunoassay kit from R&D System (Minneapolis, MN, USA).

### 4.5. Tissue Preparation and Staining

Kidneys were dissected, dehydrated, embedded in paraffin, cut into 3-μm-thick sections and prepared for periodic acid-Schiff (PAS) and Masson’s trichrome staining. An investigator blinded to the treatment group calculated the areas of glomeruli (>30 per mouse) stained positive for PAS or trichrome using an Olympus BX50 microscope with a plan objective, combined with an Olympus DP70 digital camera (Tokyo, Japan) and WinROOF image processing software (Mitani Corp., Fukui, Japan).

### 4.6. Western Blotting

Kidney tissues were homogenized in a radioimmunoprecipitation assay buffer with protease inhibitors and then centrifuged at 14,000 rpm for 30 min at 4 °C to remove debris. Protein concentration was measured by the bicinchoninic acid method. Protein extract (10 μg) was resolved using sodium dodecyl sulfate polyacrylamide gel electrophoresis, transferred to a polyvinylidene difluoride membrane and blocked using 5% non-fat milk in Tris-buffered saline with 0.1% Tween-20. After blocking, the membranes were washed and then incubated overnight with the primary antibody at 4 °C. After three washes, the membranes were incubated with horseradish peroxidase-conjugated secondary antibody for 2 h, washed again and then incubated with enhanced chemiluminescence solution. The fluorescent intensity of signals was quantified using ImageJ software (National Institutes of Health, Bethesda, MD, USA). Supplemental Table S1 describes antibodies used in the study. 

### 4.7. Reverse Transcription Polymerase Chain Reaction

Total RNA was extracted from kidneys using Sepasol RNA I super G (Nacalai). All RNA samples had a 260/280 nm ratio between 1.8 and 2.0. Reverse transcription was performed with oligo-dT primers, and the DNA was then amplified by PCR. Supplemental Table S2 describes the sequences of the primers. The PCR products were separated on a 1.5% agarose gel containing 0.01% ethidium bromide, and the intensity of the stained bands was quantitated with ImageJ software (National Institutes of Health, Bethesda, MD, USA). The amount of mRNA was normalized to the expression of glyceraldehyde-3-phosphate dehydrogenase.

### 4.8. Ethical Statement

The Mie University Committee for Animal Investigation approved the protocol of the study (Approval number 29–23; date: 15 January 2018) and the experimental procedures were performed following the institutional guidelines and internationally approved principles of laboratory animal care (NIH publication no. 85–23, revised 1985; http://grants1.nih.gov/grants/olaw/references/phspol.htm). Written informed consent was obtained from the patient.

### 4.9. Statistical Analysis

Data are expressed as the mean ± standard deviation (SD). The statistical difference between variables was calculated by Student *t*-test. Statistical analyses were done using the GraphPad Prism package software for Windows (GraphPad Software Inc., La Jolla, CA, USA). Statistical significance was considered as *p* < 0.05.

## Supplementary Materials

Supplementary materials can be found at http://www.mdpi.com/1422-0067/19/10/2902/s1.
