# Supplementary material for: Renal Injury during Long-Term Crizotinib Therapy
Source: Int J Mol Sci. 2018 Sep 25;19(10):2902. doi: 10.3390/ijms19102902 (PMC6213486; doi:10.3390/ijms19102902)
Supplement: Supplementary file 1 [file ijms-19-02902-s001.pdf]

Supplemental Materials

Renal Injury during Long-term Crizotinib Therapy

Taro Yasuma, Tetsu Kobayashi, Corina N. D'Alessandro-Gabazza, Hajime Fujimoto, Kentaro Ito, Yoichi Nishii, Kota Nishihama, Prince Baffour Tonto, Atsuro Takeshita, Masaaki Toda, Esteban C. Gabazza, Osamu Taguchi, Shigenori Yonemura, Osamu Hataji.

Supplementary Table 1. Antibodies used in the experimental mouse model

Antibodies	Catalog No	Host and type	Company (city, state /country)
Primary antibodies			
Anti-phospho-c-MET	AF2480	Rabbit polyclonal IgG	R&D System (Minneapolis, MN)
Anti-c-MET	AF527	Goat polyclonal IgG	R&D System (Minneapolis, MN)
Anti-phospho-STAT3	sc-8059	Mouse monoclonal IgG	Santa Cruz (Santa Cruz, CA)
Anti-STAT3	sc-8019	Mouse monoclonal IgG	Santa Cruz (Santa Cruz, CA)
Anti-phospho-ERK1/2	sc-7383	Mouse monoclonal IgG	Santa Cruz (Santa Cruz, CA)
Anti-ERK1/2	sc-514302	Mouse monoclonal IgG	Santa Cruz (Santa Cruz, CA)
Anti-phospho-Smad2	#3104	Rabbit polyclonal IgG	Cell Signaling Technology (Danvers, MA)
Anti-Smad2	#3122	Rabbit monoclonal IgG	Cell Signaling Technology (Danvers, MA)
Anti-phospho-IkB	#4792	Rabbit monoclonal IgG	Cell Signaling Technology (Danvers, MA)
Anti-IkB	#4812	Rabbit monoclonal IgG	Cell Signaling Technology (Danvers, MA)
Anti-phospho-AKT	sc-514032	Mouse monoclonal IgG	Santa Cruz (Santa Cruz, CA)
Anti-AKT	sc-81434	Mouse monoclonal IgG	Santa Cruz (Santa Cruz, CA)
Anti-beta Actin	#4970	Rabbit monoclonal IgG	CST, (Danvers, MA)
Secondary antibodies			
Anti-rabbit IgG-HRP	#172-1019	Goat polyclonal IgG	Bio Rad (Hercules, CA)
Anti-goat IgG-HRP	#172-1034	Rabbit polyclonal IgG	Bio Rad (Hercules, CA)
Anti-mouse IgG-HRP	#170-6516	Goat polyclonal IgG	Bio Rad (Hercules, CA)

Supplementary Table 2. Sequence of PCR primers

Gene	Direction	Sequence (5’ to 3’)	Reference	Location	Amplicon size
mGapdh	sense	TGGCCTTCCGTGTTCCTAC	NM 008084	686-704	178 bp
	antisense	GAGTTGCTGTTGAAGTCGCA 20	863-844		
mIL-6	sense	CTGGTCTTCTGGAGTACCATAG	NM 031168	375-396	374 bp
	antisense	AAGTCAGATACCTGACAACAGG	748-727		
mTNF-α	sense	ACGTGGAACTGGCAGAAGAG	NM 013693	182-201	284 bp
	antisense	CTCCTCCACTTGGTGGTTTG 20	465-446		
mTGF-β1	sense	ACTCCACGTGGAAATCAACGG	NM 011577	1560-1580	414 bp
	antisense	TAGTAGACGATGGGCAGTGG 20	1973-1954		
mMMP-2	sense	CACCACCGAGGACTATGACC 20	NM 008610	954-973	122 bp
	antisense	TGTTGCCCAGGAAAGTGAAG 20	1075-1056		
mCol1a1	sense	TAAGGGTCCCCAATGGTGAGA	NM 007742	107-127	203 bp

PCR, polymerase chain reaction; m, mouse; Gapdh, Glyceraldehyde 3-phosphate dehydrogenase; IL-6, interleukin-6; TNF-α, tumor necrosis factor-α; TGF-β1, transforming growth factor-β1; MMP2, matrix metalloproteinase-2.
